# Supplementary material for: Six-photon upconverted excitation energy lock-in for ultraviolet-C enhancement
Source: Nat Commun. 2021 Jul 16;12:4367. doi: 10.1038/s41467-021-24664-x (PMC8285497; doi:10.1038/s41467-021-24664-x)
Supplement: Supplementary file 1 — Supplementary Information [file 41467_2021_24664_MOESM1_ESM.pdf]

Supplementary Material:

Six-Photon Upconverted Excitation Energy Lock-in for Ultraviolet-C Enhancement

Qianqian Su[1,†,*], Han-Lin Wei[1,†], Yachong Liu[1], Chaohao Chen[2], Ming Guan[3],Shuai Wang[1], Yan Su[4], Haifang Wang[1,*], Zhigang Chen[5], and Dayong Jin[2,3,*]

[1]Institute of Nanochemistry and Nanobiology, Shanghai University, Shanghai 200444, China

[2]Institute for Biomedical Materials & Devices (IBMD), Faculty of Science, University of Technology, Sydney, Ultimo, New South Wales 2007, Australia.

[3]UTS-SUStech Joint Research Centre for Biomedical Materials & Devices, Department of Biomedical Engineering, Southern University of Science and Technology, Shenzhen, Guangdong 518055, P. R. China.

[4]Genome Institute of Singapore, Agency of Science Technology and Research, 138672, Singapore.

[5]State Key Laboratory for Modification of Chemical Fibers and Polymer Materials, College of Materials Science and Engineering, Donghua University, Shanghai 201620, China.

*To whom correspondence should be addressed, e-mail: Q.S. (chmsqq@shu.edu.cn), H.W. (hwang@shu.edu.cn), or D.J. (dayong.jin@uts.edu.au).

†These authors contributed equally.

Supplementary Figures

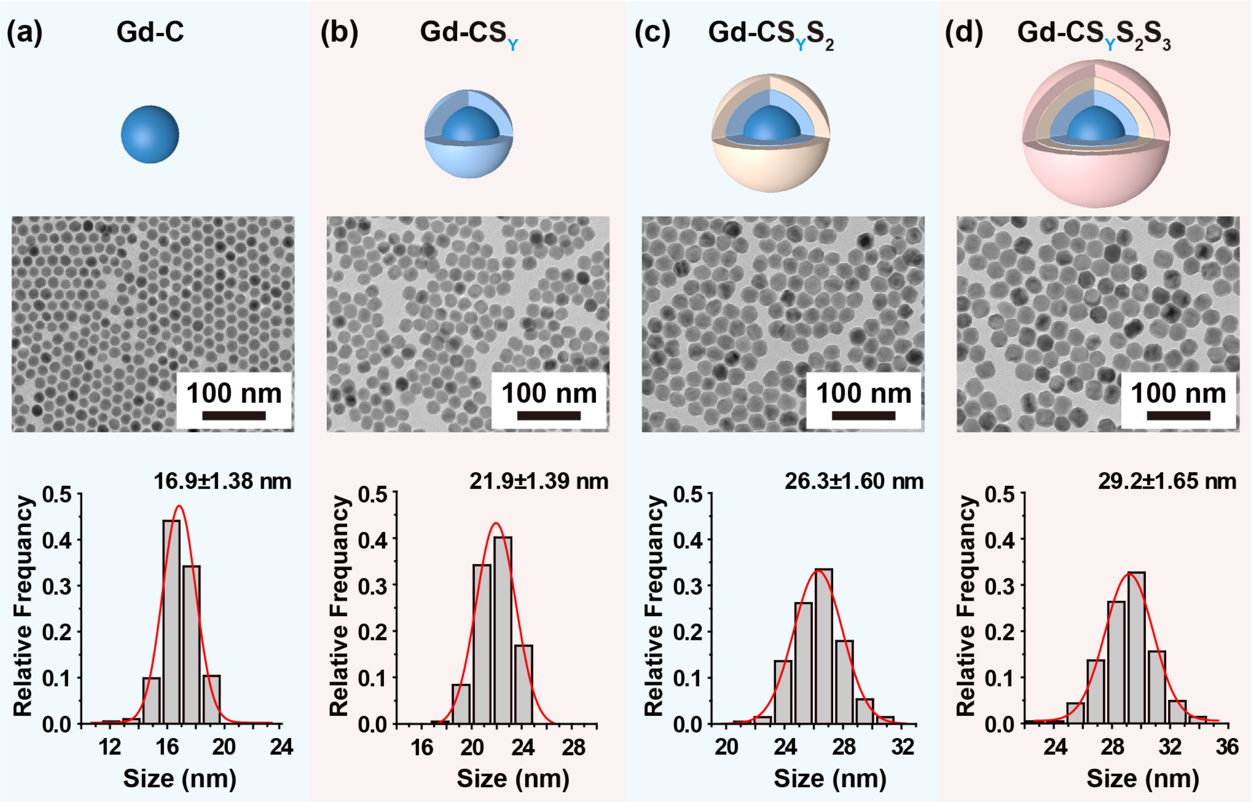

Supplementary Figure 1. Transmission electron microscopy (TEM) images and size distributions of the as-synthesized $NaGdF_4$:49%Yb,1%Tm@$NaYF_4$:20%Yb@$NaGdF_4$:10%Yb,50%Nd@$NaGdF_4$ (Gd-$CS_YS_2S_3$) core (a), core-shell (b), core-shell-shell (c), and core-multishell (d) nanoparticles. Nanoparticle size distribution is fitted by a Gaussian curve (red full line).

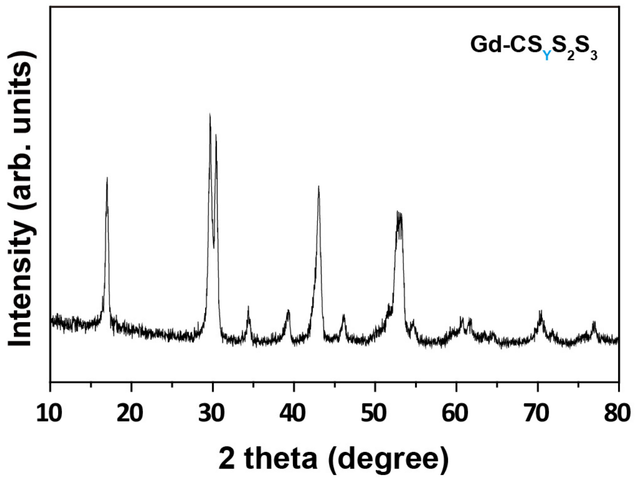

Supplementary Figure 2. X-ray powder diffraction (XRD) pattern of the as-synthesized heterogeneous core-multishell nanoparticles.

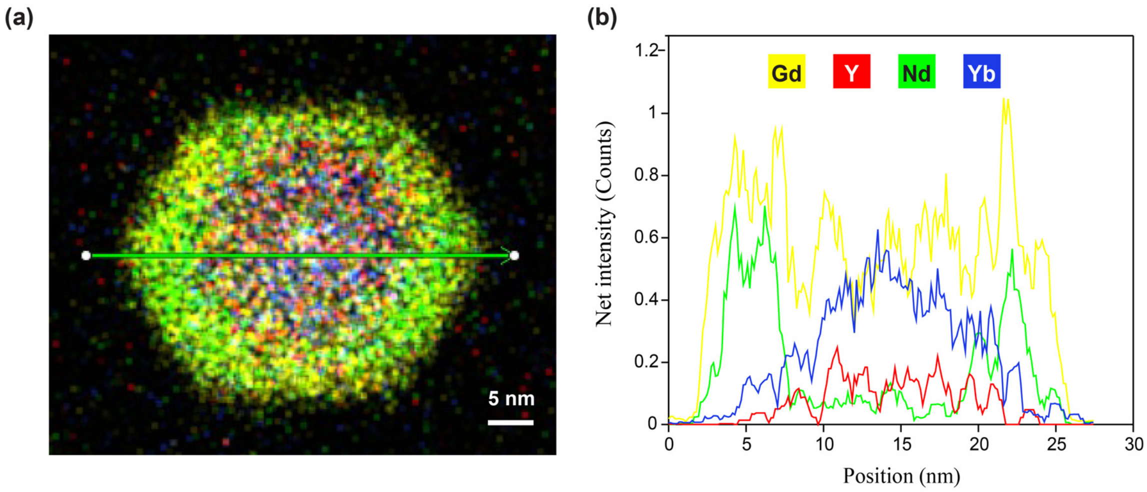

Supplementary Figure 3. (a) Elemental mapping of a single Gd-$CS_YS_2S_3$ nanoparticle. (b) Energy-dispersive X-ray mapping analysis of the heterogeneous core-multishell nanoparticle.

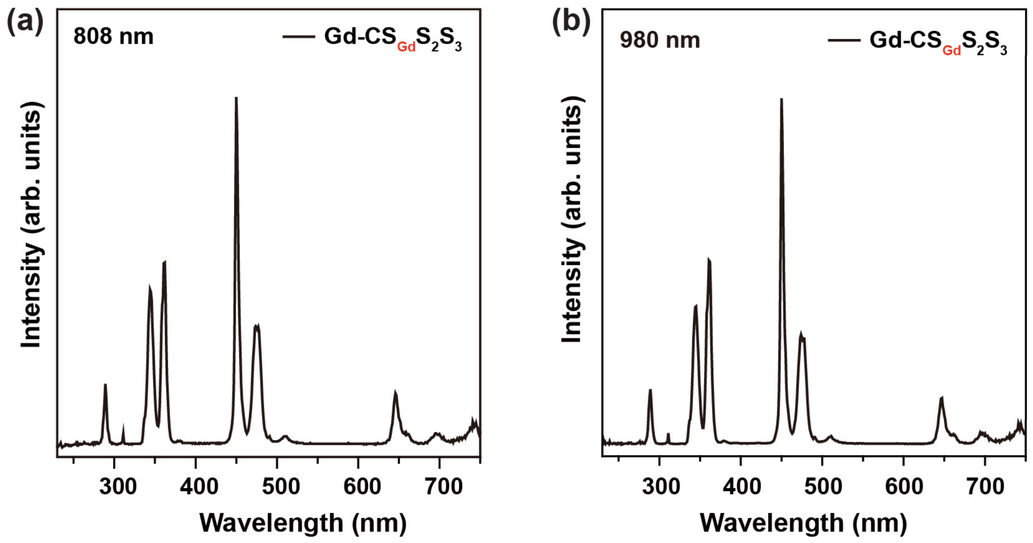

Supplementary Figure 4. Emission spectra of Gd-$CS_{Gd}S_2S_3$ nanoparticles under excitation of 808 nm (a) and 980 nm (b).

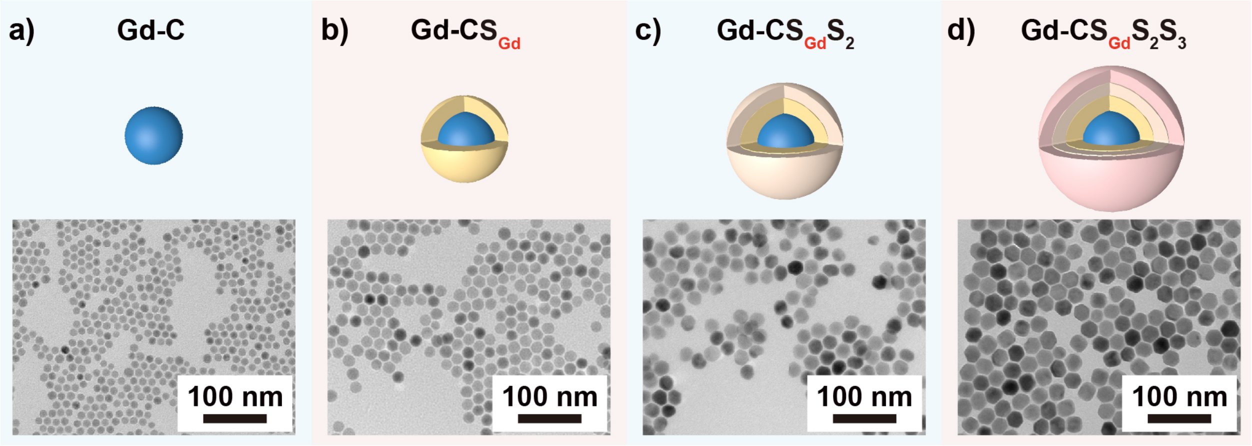

Supplementary Figure 5. TEM images of the as-synthesized conventional core-multishell nanoparticles $NaGdF_4$:49%Yb,1%Tm@$NaGdF_4$:20%Yb@$NaGdF_4$:10%Yb,50%Nd@$NaGdF_4$ (Gd-$CS_{Gd}S_2S_3$), core (a), core-shell (b), core-shell-shell (c), and core-multishell (d) nanoparticles.

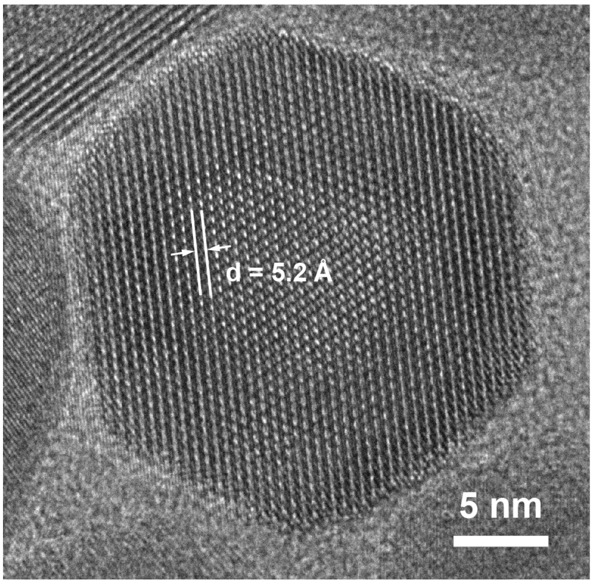

Supplementary Figure 6. High-resolution TEM (HRTEM) image of an as-synthesized conventional core-multishell Gd-$CS_{Gd}S_2S_3$ nanoparticle.

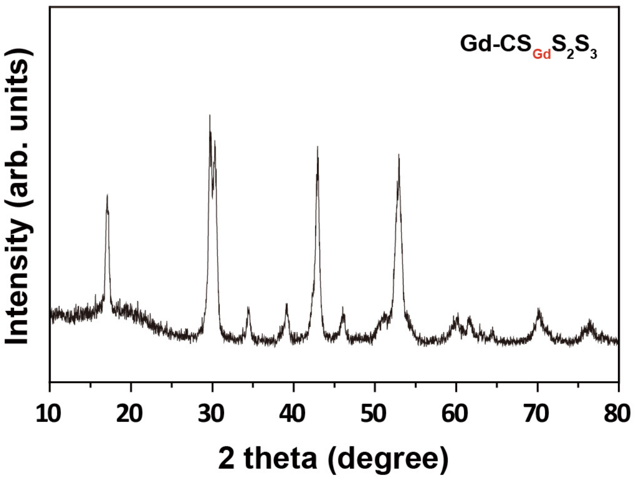

Supplementary Figure 7. XRD pattern of the as-synthesized heterogeneous core-multishell nanoparticles.

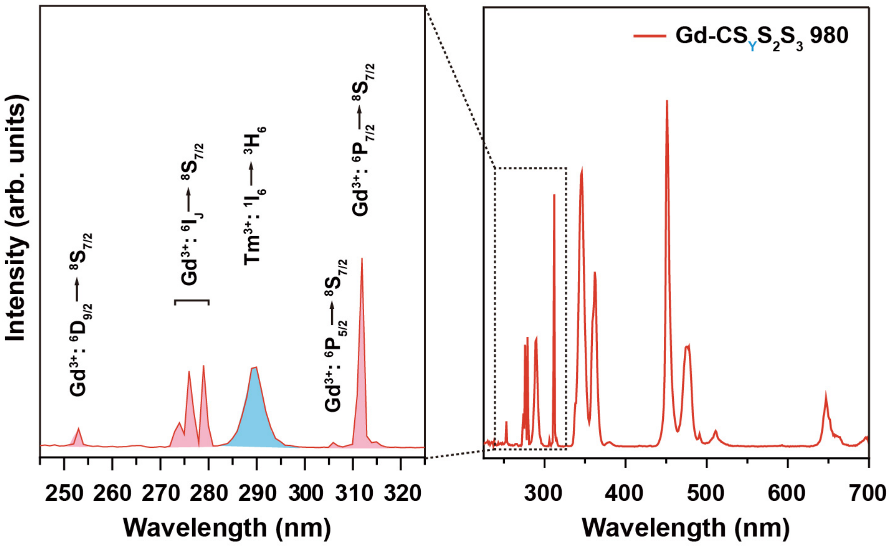

Supplementary Figure 8. The emission spectra of the as-synthesized core-multishell nanoparticles Gd-$CS_YS_2S_3$ under 980 nm excitation.

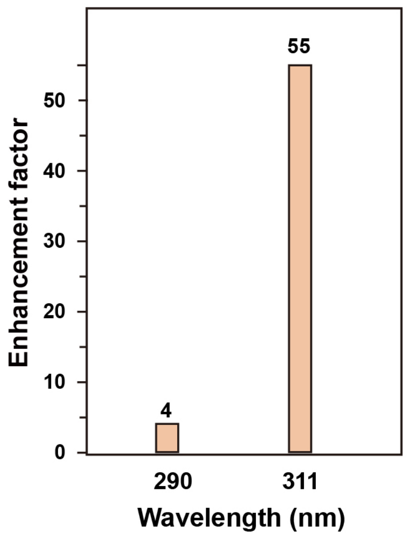

Supplementary Figure 9. The enhancement factor of the emission at 290 and 311 nm obtained by comparing the results for Gd-$CS_YS_2S_3$ and Gd-$CS_{Gd}S_2S_3$ nanoparticles under excitation of 808 nm CW diode laser. The excitation power density is 10 W cm^{-2}.

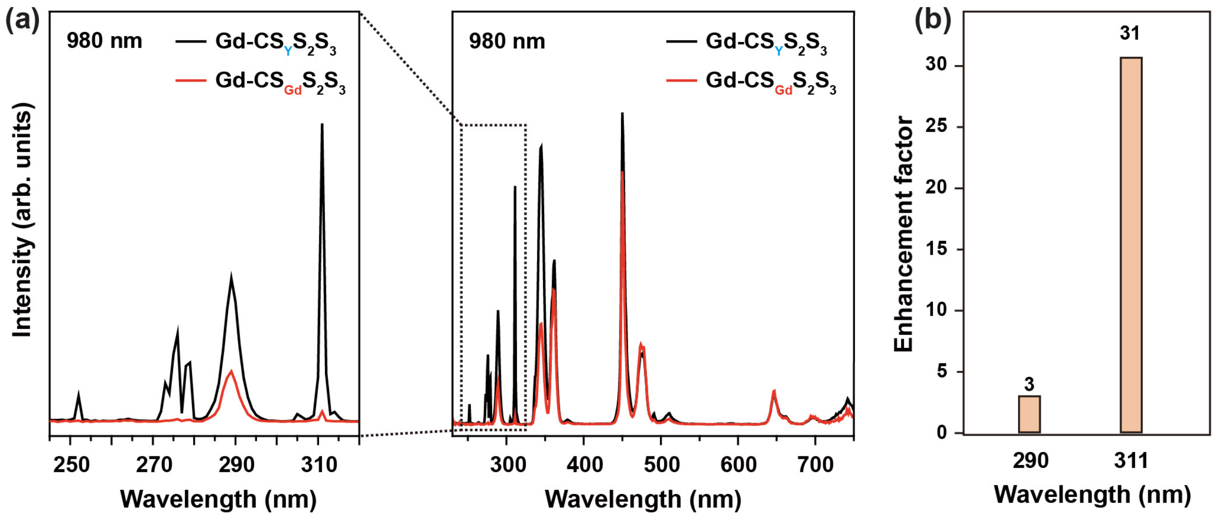

Supplementary Figure 10. (a) Emission spectra of Gd-CS$_Y$S$_2$S$_3$ and Gd-CS$_{Gd}$S$_2$S$_3$ nanoparticles with different migration layer host matrices under excitation of 980 nm. (b) The enhancement factor of the emission at 290 and 311 nm obtained by comparing the results for Gd-CS$_Y$S$_2$S$_3$ and Gd-CS$_{Gd}$S$_2$S$_3$ nanoparticles under excitation of 980 nm CW diode laser. The excitation power density is 10 W cm^{-2}.

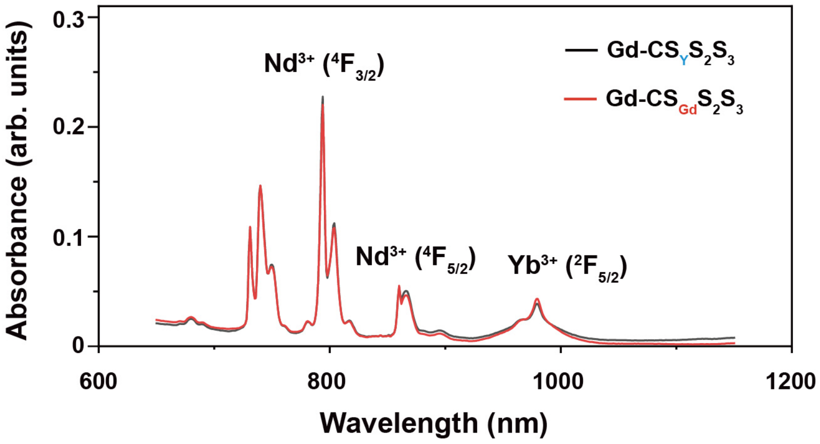

Supplementary Figure 11. Absorption spectra of Gd-$CS_YS_2S_3$ and Gd-$CS_{Gd}S_2S_3$ nanoparticles.

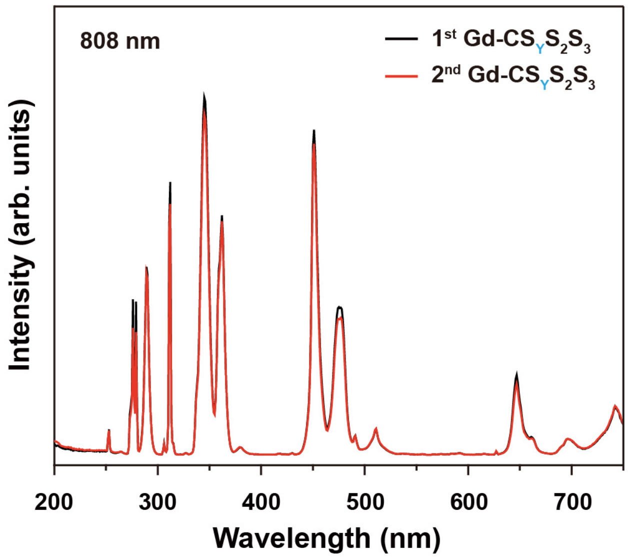

Supplementary Figure 12. Emission spectra of two batches of the as-synthesized heterogeneous core-multishell nanoparticles under 808 nm excitation, demonstrating the synthesis of ultraviolet-C (UVC) upconversion nanoparticles is repeatable.

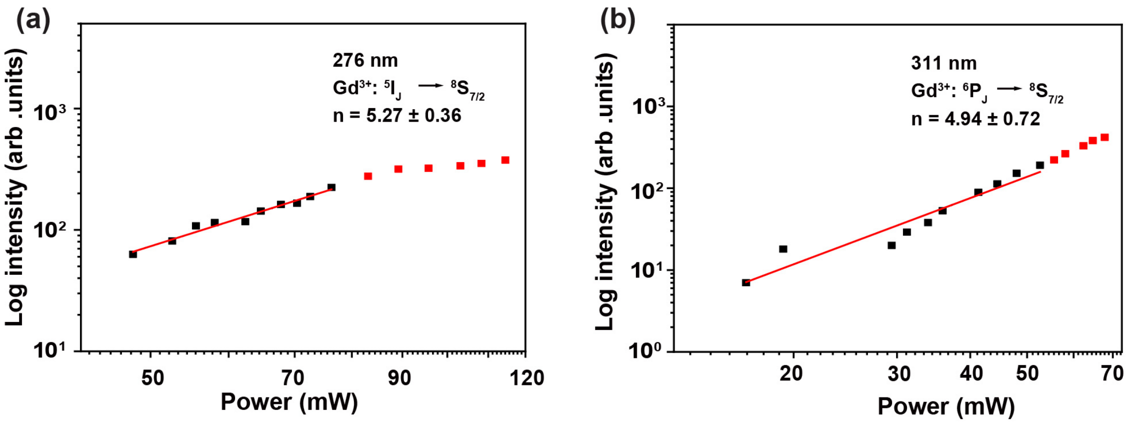

Supplementary Figure 13. (a,b) Log intensity-pump power plots of the 276 and 311 nm upconversion emission of Gd-$C_YS_2S_3$ nanoparticles under 808 nm excitation, respectively.

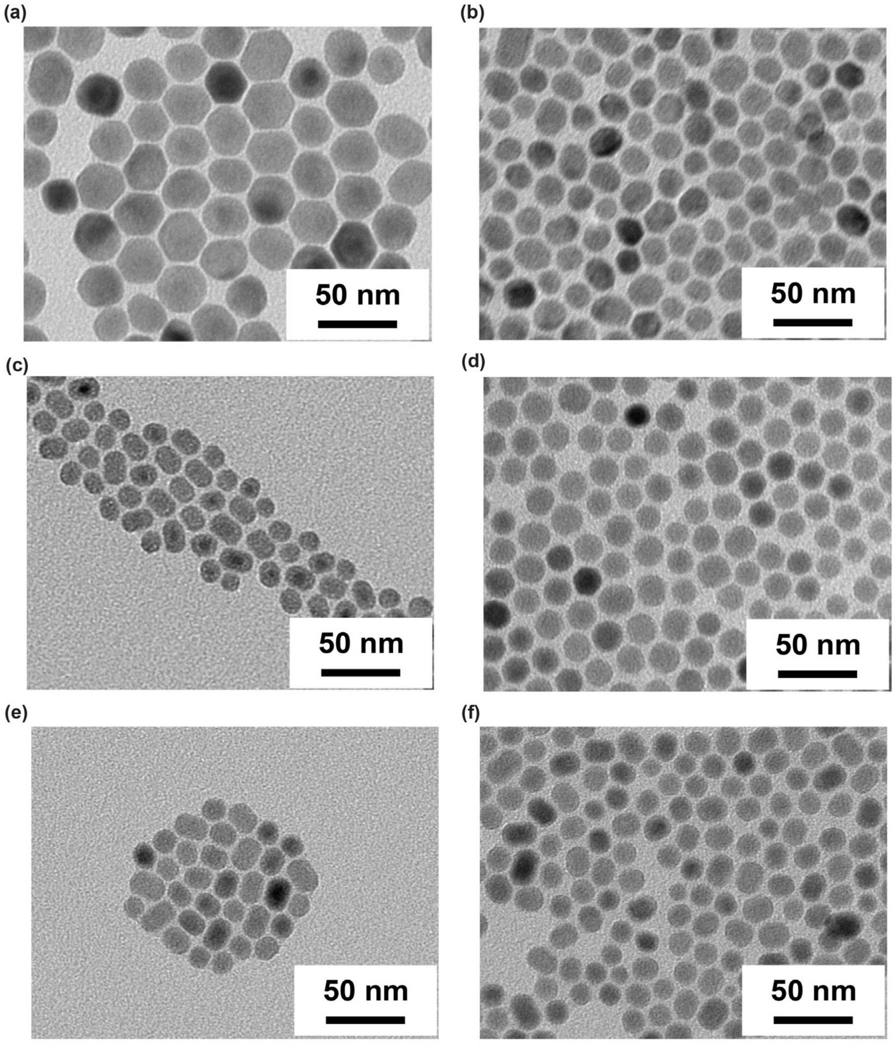

Supplementary Figure 14. TEM images of the as-synthesized nanoparticles $NaGdF_4$:49%Yb,1%Tm@$NaYF_4$:20%Yb@$NaGdF_4$:10%Yb,50%Nd@$NaGdF_4$ (a), $NaGdF_4$:49%Y,1%Tm@ $NaYF_4$@$NaGdF_4$:10%Y,50%Nd@$NaGdF_4$ (b), $NaGdF_4$:20%Yb,1%Tm,29%Y@$NaYF_4$ (c), $NaGdF_4$:20%Yb,30%Y@$NaYF_4$ (d), $NaGdF_4$:20%Yb,1%Tm,29%Y@$NaYF_4$ (e), and $NaYF_4$: 20%Yb,1%Tm@$NaYF_4$ (f).

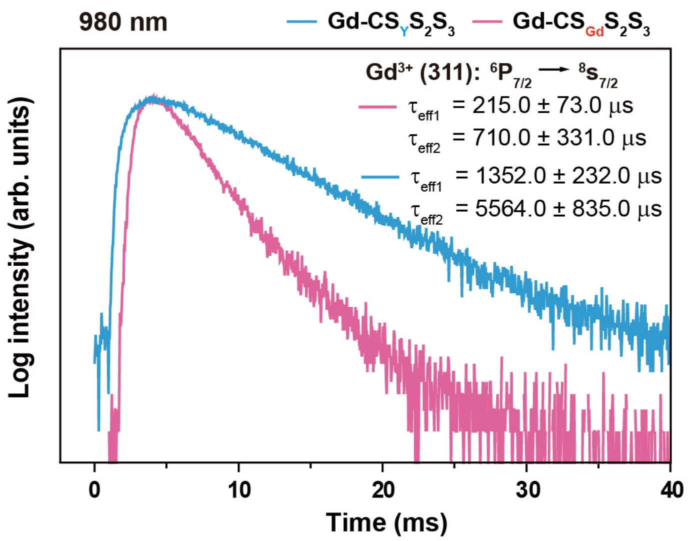

Supplementary Figure 15. Upconversion luminescence decay curves of Gd^{3+} emissions at 311 nm of Gd-CS$_{Gd}$S$_2$S$_3$ and Gd-CS$_Y$S$_2$S$_3$ nanoparticles under 980 nm excitation.

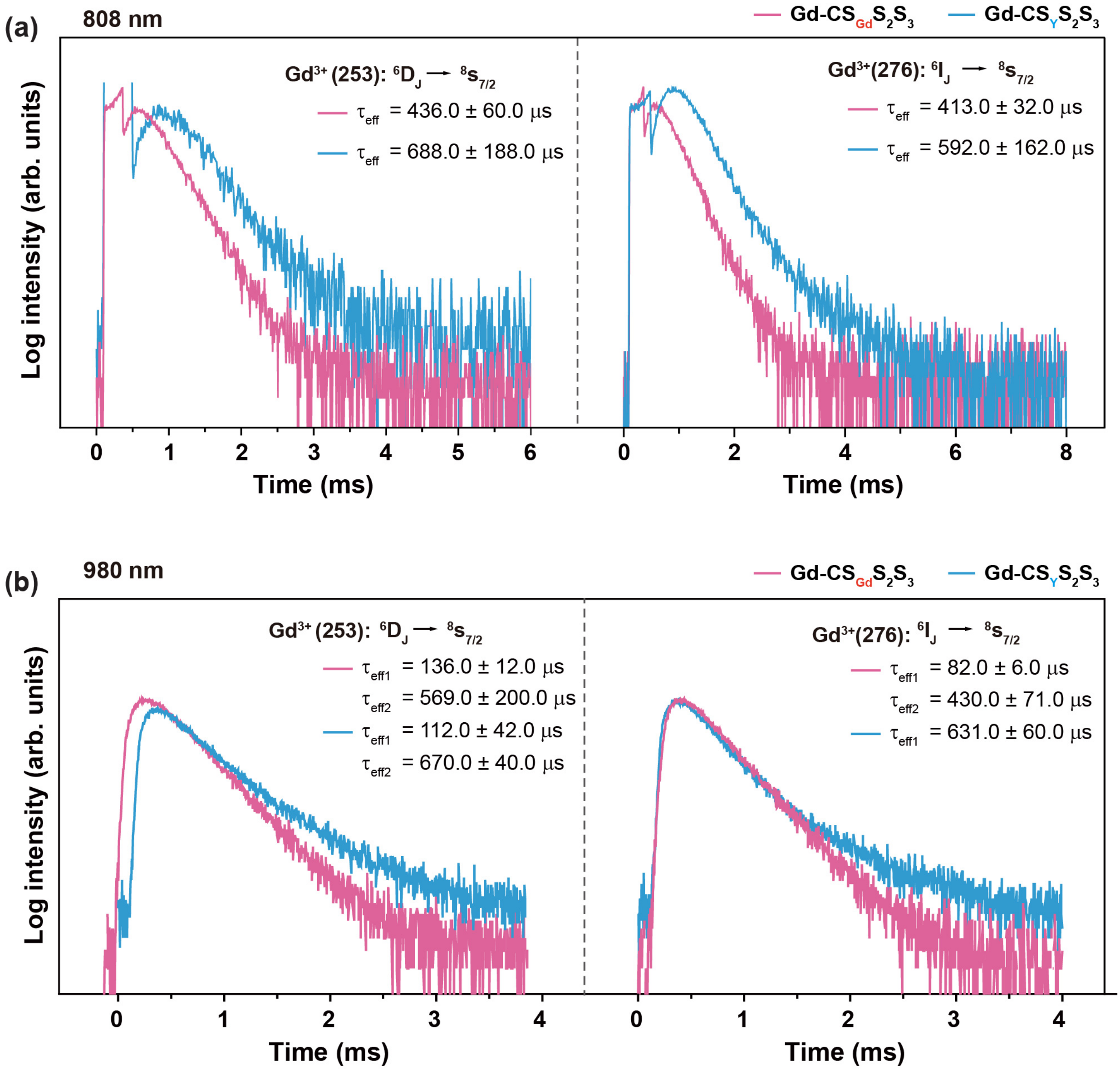

Supplementary Figure 16. Upconversion luminescence decay curves of Gd^{3+} emissions of Gd-CS$_{Gd}$S$_2$S$_3$ and Gd-CS$_Y$S$_2$S$_3$ nanoparticles at 253 and 276 nm under 808 nm (a) and 980 nm (b) excitation

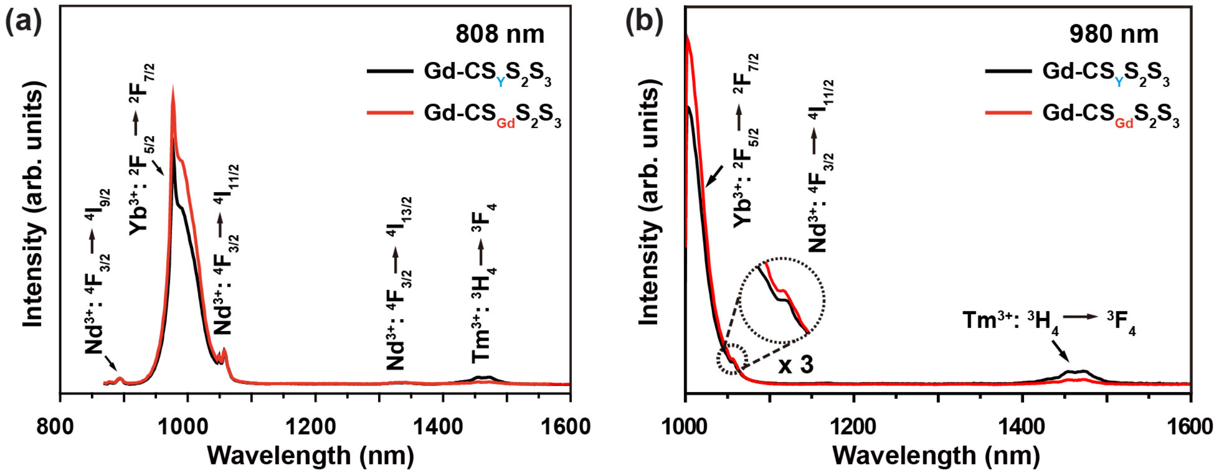

Supplementary Figure 17. Emission spectra of Gd-$CS_{Gd}S_2S_3$ and Gd-$CS_YS_2S_3$ nanoparticles in near infrared range under 808 and 980 nm excitation.

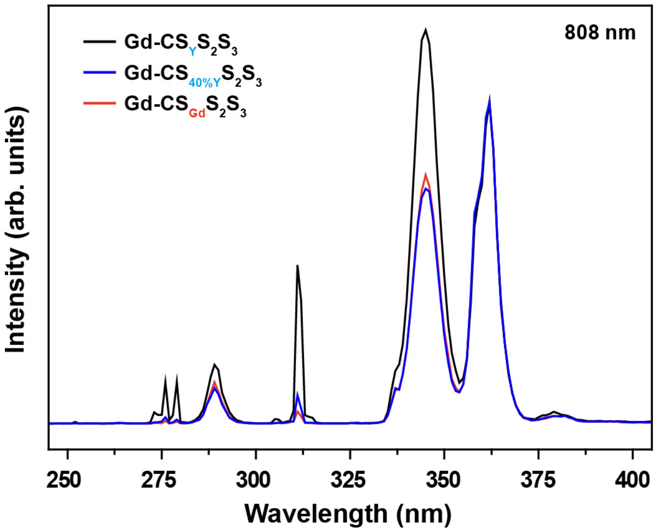

Supplementary Figure 18. Emission spectra of Gd-CS$_Y$S$_2$S$_3$, Gd-CS$_{40\%Y}$S$_2$S$_3$ and Gd-CS$_{Gd}$S$_2$S$_3$ nanoparticles under 808 nm excitation.

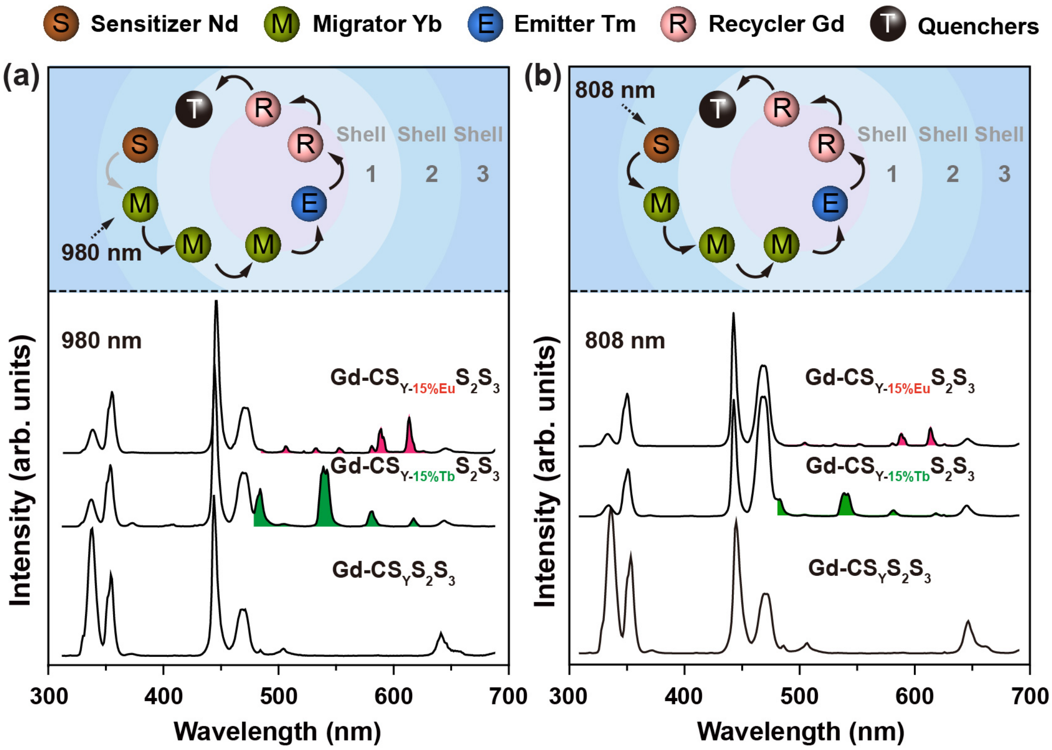

Supplementary Figure 19. (a, b) Upconversion luminescence spectra curves of Gd-CS$_Y$S$_2$S$_3$ with and without Tb^{3+} and Eu^{3+} doping in the first layer under 980 nm and 808 nm excitation.

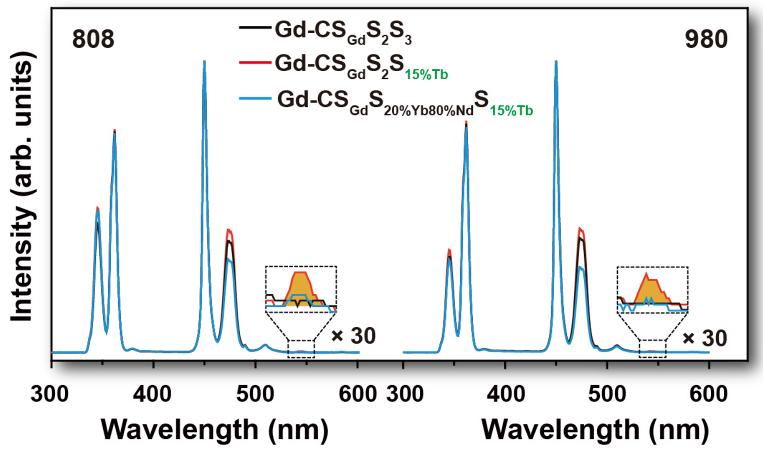

Supplementary Figure 20. Upconversion emission spectra of Gd-CS$_{Gd}$S$_2$S$_3$, Gd-CS$_{Gd}$S$_2$S$_{15\%Tb}$, and Gd-CS$_{Gd}$S$_{20\%Yb80\%Nd}$S$_{15\%Tb}$ nanoparticles, suggesting only a very small amount of excitation energy was leaked from Gd^{3+} to the surface of nanoparticles.

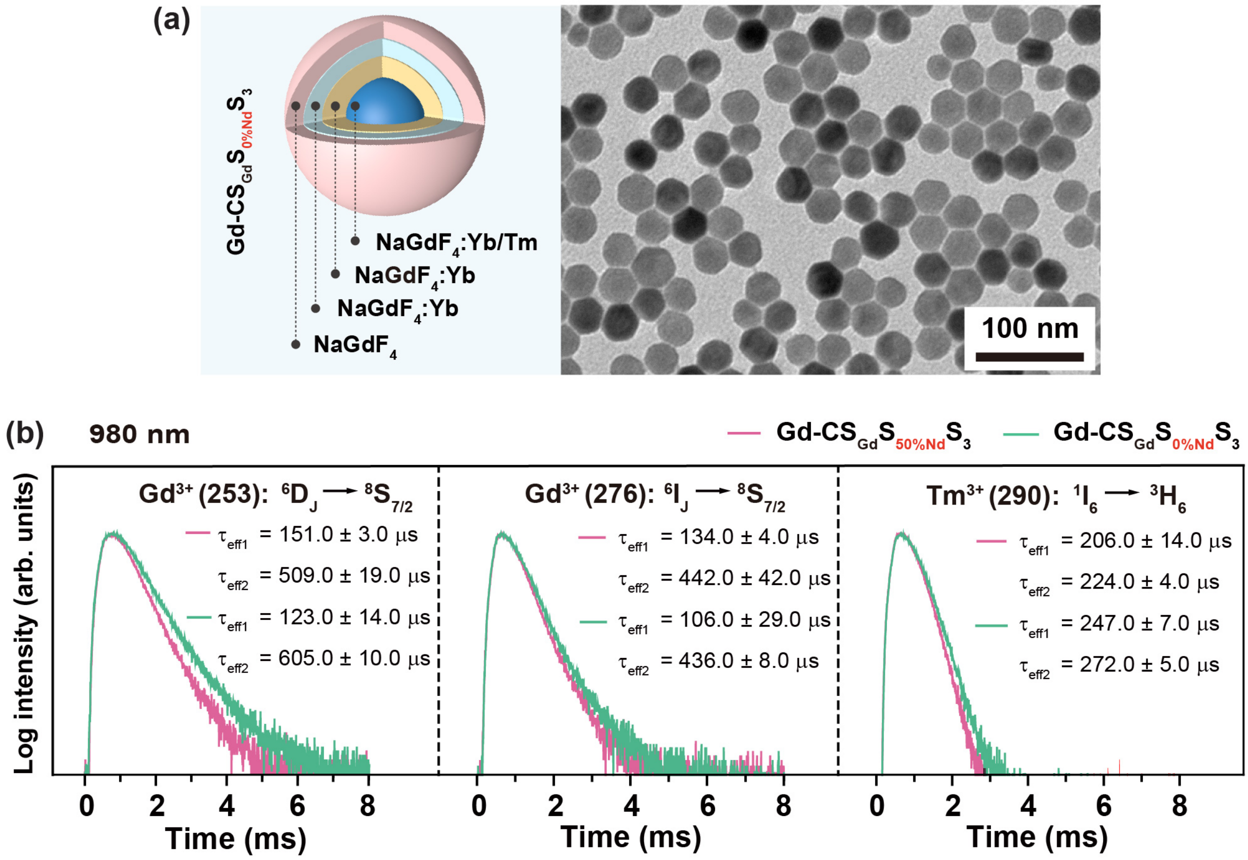

Supplementary Figure 21. (a) Schematic illustration of the as-synthesized Gd-CS$_{Gd}$S$_{0\%Nd}$S$_3$ nanoparticles and their TEM image. (b) Upconversion luminescence decay curves of Gd^{3+} emissions at 253 and 276 nm and Tm^{3+} emission at 290 nm of Gd-CS$_{Gd}$S$_{50\%Nd}$S$_3$ and Gd-CS$_{Gd}$S$_{0\%Nd}$S$_3$ nanoparticles under 980 nm excitation, respectively.

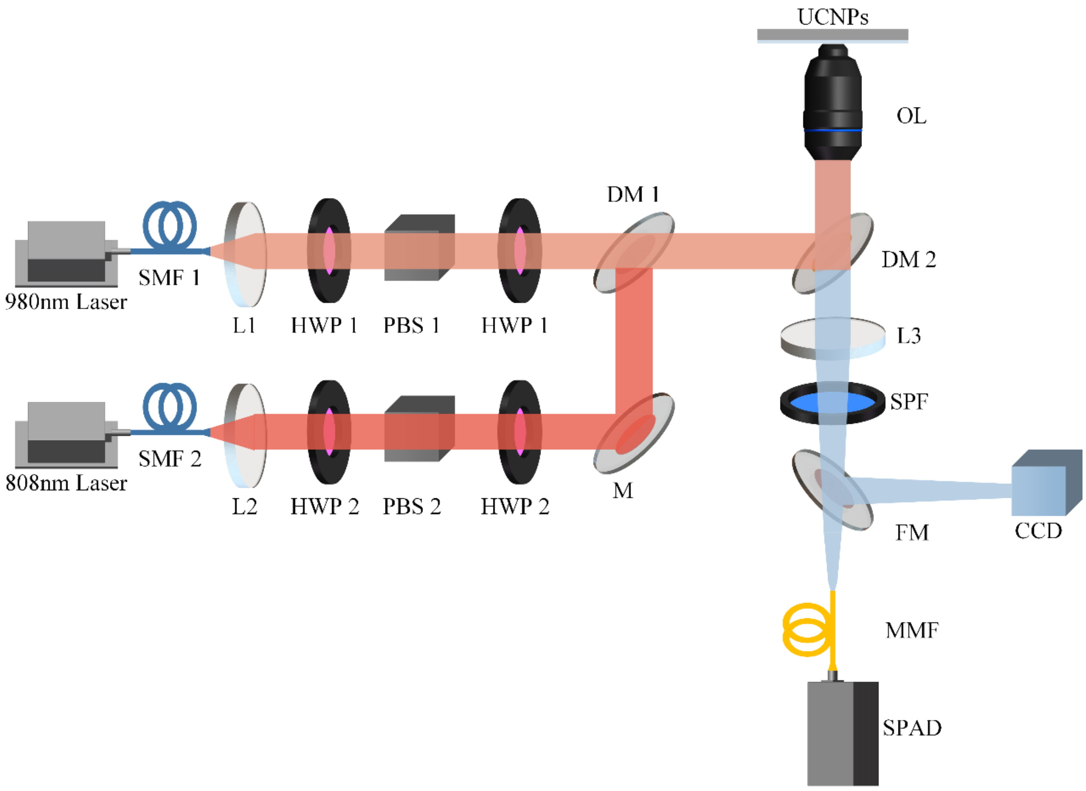

Supplementary Figure 22. Experimental setup for confocal microscopy (SMF, single-mode fiber; L1 & L2, collimation lens; L3, collection len; HWP, half-wave plate; PBS, polarized beam splitter; M, mirror; FM, flexible mirror; DM, dichroic mirror; OL, objective len; SPF, 750 nm short pass filter; SPAD, single-photon avalanche diode; CCD, charge coupled device).

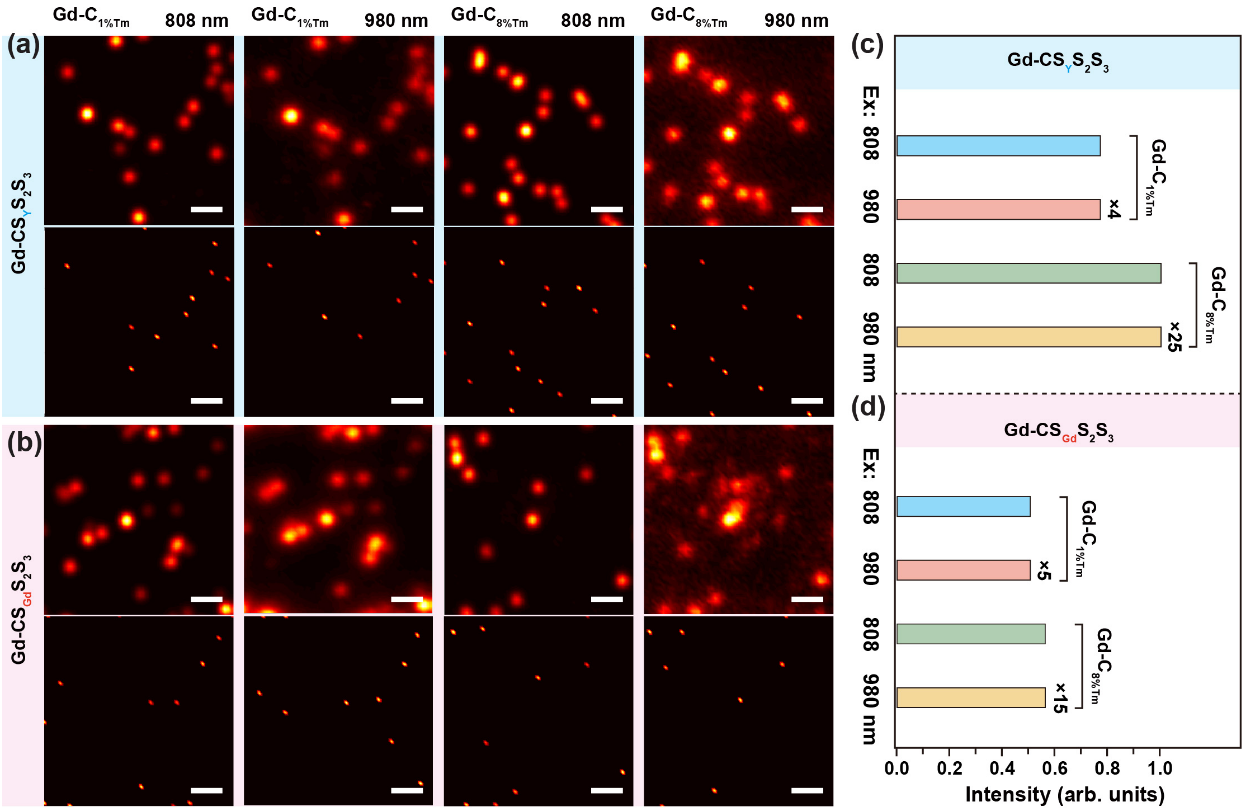

Supplementary Figure 23. (a) Nanoparticle images (400 nm to 750 nm emissions) and the processed images of selected single nanoparticles in the predefined region (bottom) of Gd-CS$_Y$S$_2$S$_3$ doped with 1% Tm and 8% Tm under 808 nm and ~980 nm excitation. (b) Nanoparticle images (400 nm to 750 nm emissions) and the processed images of selected single nanoparticles in the predefined region (bottom) Gd-CS$_{Gd}$S$_2$S$_3$ doped with 1% Tm and 8% Tm under 808 nm and ~980 nm excitation. The scale bar represents 1 m. (c-d) Comparison of signal intensities of Gd-CS$_Y$S$_2$S$_3$ and Gd-CS$_{Gd}$S$_2$S$_3$ doped with 1% Tm and 8% Tm under 808 nm and ~980 nm excitation.

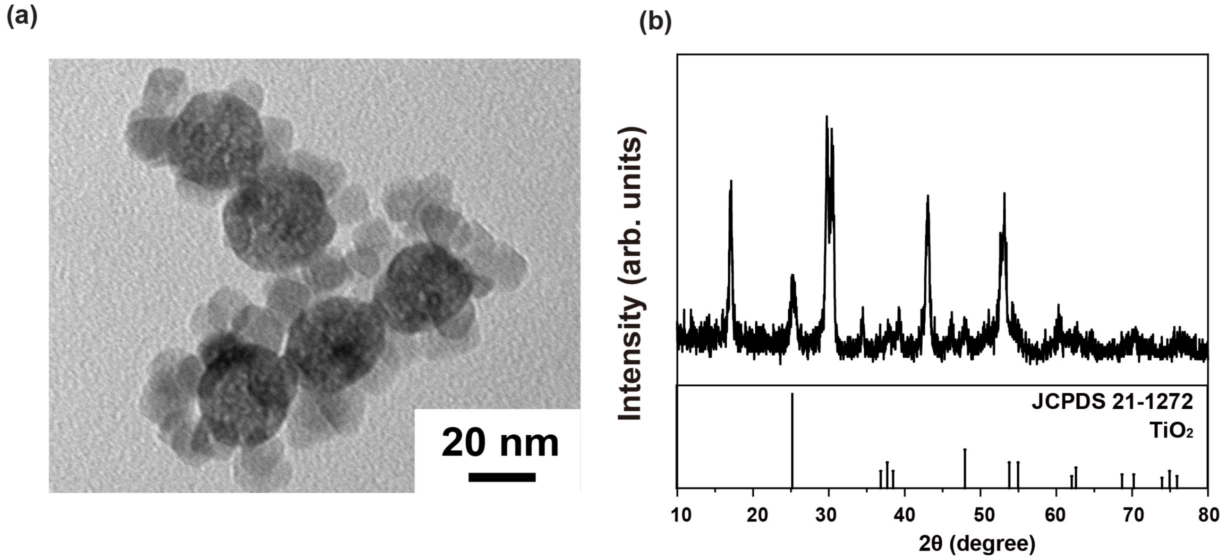

Supplementary Figure 24. (a) TEM image of the as-synthesized Gd-$CS_YS_2S_3$@TiO_2 nanocompoties. (b) XRD pattern of the as-synthesized Gd-$CS_YS_2S_3$@TiO_2 nanocompoties.

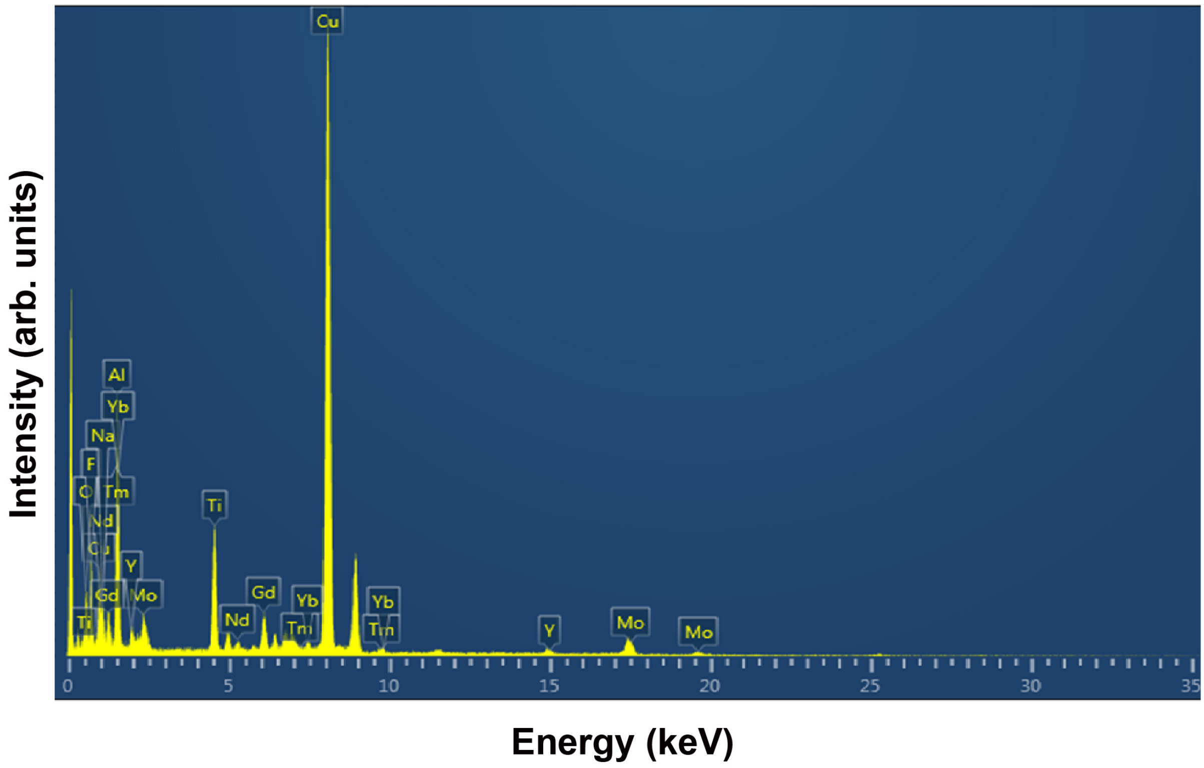

Supplementary Figure 25. Energy dispersive X-ray (EDX) spectrum of Gd-$CS_YS_2S_3$@TiO_2 nanocompoties.

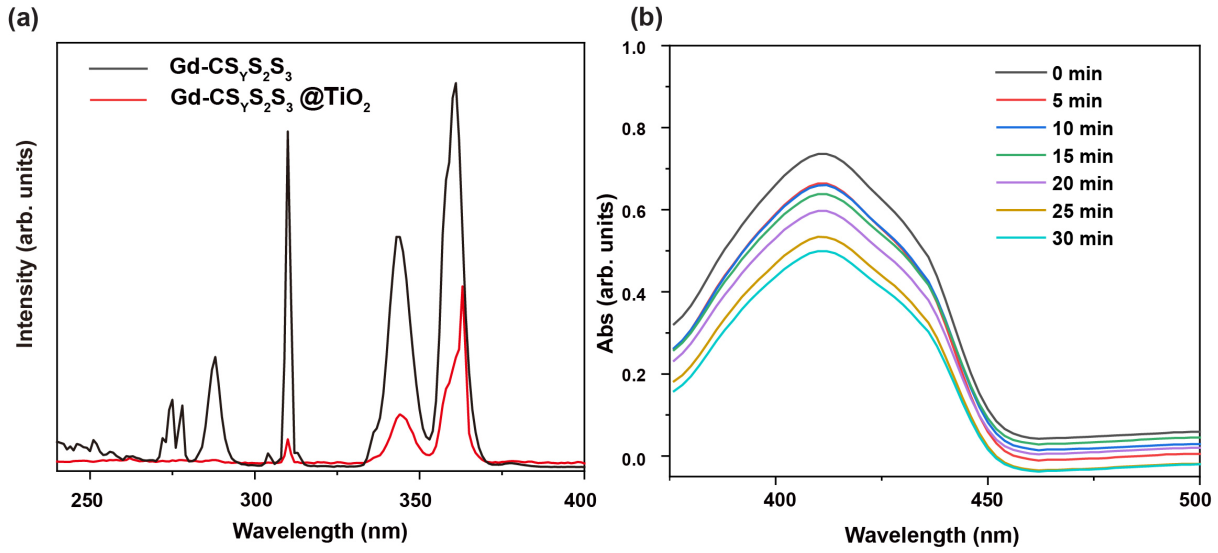

Supplementary Figure 26. (a) Emission spectra of Gd-CS$_Y$S$_2$S$_3$ and Gd-CS$_Y$S$_2$S$_3$@TiO$_2$ under 808 nm excitation. (b) Absorbance changes of DPBF treated with Gd-CS$_Y$S$_2$S$_3$@TiO$_2$ nanocompoties accompanied with 808 nm laser irradiation for different times.

Supplementary Methods

Experimental Procedures

Materials. $Gd(CH_3CO_2)_3·xH_2O$ (99.9%), $Nd(CH_3CO_2)_3·xH_2O$ (99.9%), $Y(CH_3CO_2)_3·xH_2O$ (99.9%), $Yb(CH_3CO_2)_3·xH_2O$ (99.9%), $Tm(CH_3CO_2)_3·xH_2O$ (99.9%), $Tb(CH_3CO_2)_3·xH_2O$ (99.9%), $Eu(CH_3CO_2)_3·xH_2O$ (99.9%), $Er(CH_3CO_2)_3·xH_2O$ (99.9%), $Ho(CH_3CO_2)_3·xH_2O$ (99.9%), NaOH (>98%), NH_4F (>98%), oleic acid (OA, 90%), and 1-octadecene (ODE, 90%), 1,3-diphenylisobenzofuran (DPBF, 97%), TiF_4, polyvinylpyrrolidone (PVP, Mw = 40,000) were all purchased from Sigma-Aldrich. Cyclohexane (AR) and ethanol (AR) were purchased from Sinopharm Chemical Reagent Co., Ltd. All chemicals were used as received without further purification.

Characterization. Luminescence spectra were measured at room temperature with an FS5 or FLS1000 (Edinburgh) in conjunction with 808 nm (cnilaser, MDL-III-808-2.5W, China) and 980 nm (cnilaser, MDL-III-980-2.0W, China) diode lasers or OPO laser (Opolette 355, Opotek) at 778 nm. Quantum yields were recorded at room temperature with an FLS1000 equipping with integrating sphere (Edinburgh). The decay curves were recorded with ultraviolet to near-infrared steady-state and phosphorescence lifetime spectrometer (FS5 or FLS1000, Edinburgh), in conjunction with pulsed 808 nm, 980 nm diode lasers, or OPO laser (Opolette 355, Opotek) at 920 nm. Transmission electron microscopy (TEM) measurements were carried out on an HT7700 field emission transmission electron microscope operated at an acceleration voltage of 120 kV. Energy dispersive X-ray (EDX) spectrum was carried out on an HT7700 field emission transmission electron microscope equpping with Oxford Instruments. High-resolution TEM images were carried out using an FEI Talos F200S transmission electron microscope operated at an acceleration voltage of 200 kV. HAADF-STEM and elemental mapping images were performed with an FEI Talos F200X transmission electron microscope. Powder X-ray diffraction (XRD) analysis was performed on a Rigaku D/MAX-2200 equipped with a rotating anode and a Cu Kα radiation source (λ = 0.15418 nm). The excitation power density was measured by laser power densitometer TS5 (Changchun New Industries Optoelectronics Technology, China). UV-vis absorption spectra were obtained by using a PerkinElmer Lambda 750 ultraviolet-visible-near infrared spectrometer and Hitachi U-3010. All spectra were recorded under identical experimental conditions unless otherwise noted. Key experiments were repeated three times and all other experiments were repeated twice.

Synthesis of $NaGdF_4$:Yb/Tm core nanoparticles. $NaGdF_4$ doped with 49%Yb/1%Tm nanoparticles were synthesized according to a modified literature procedure[1-3]. Typically, $Gd(CH_3CO_2)_3$ (0.067 g; 0.2 mmol), $Yb(CH_3CO_2)_3$ (0.069 g; 0.196 mmol) and $Tm(CH_3CO_2)_3$ (0.001 g; 0.004 mmol) were dissolved in a water

solution and combined (2 mL) in a 50 mL two-neck round-bottom flask charged with OA (5 mL) and ODE (5 mL) at room temperature. The mixture was then heated to 150 °C and kept at this temperature for 1 h. After cooling down to 50 °C, a methanol solution (4.4 mL) containing NH_4F (0.05 g; 1.36 mmol) and NaOH (0.04 g; 1 mmol) was added and stirred for 30 min. The mixture was heated to 100 °C *in vacuo* for 15 min to remove the methanol. Subsequently, the resulting solution was heated to 300 °C and kept at this temperature for 1.5 h with argon. The obtained nanoparticles were collected by centrifugation at 6000 rpm for 5 min, and then washed with ethanol and cyclohexane for three times. The core nanoparticles were dispersed in cyclohexane (4 mL) prior to being used for shell coating.

Synthesis of $NaGdF_4$:Yb/Tm@$NaYF_4$:20%Yb and $NaGdF_4$:Yb/Tm@$NaGdF_4$:20%Yb core-shell nanoparticles. The synthesis of core-shell nanoparticles followed a similar procedure to our previous report[3]. The pre-synthesized $NaGdF_4$:Yb/Tm core nanoparticles were used as seeds for shell coating. The precursor solution of the shell was first prepared via the same procedure as mentioned above and then cooled down to 80 °C. A cyclohexane dispersion of $NaGdF_4$:Yb/Tm nanoparticle seeds (4 mL) were added, and then a methanol solution of NH_4F (0.05 g; 1.36 mmol) and NaOH (0.04 g; 1 mmol) was then added and stirred at 50 °C for 30 min. The reaction was heated to 300 °C under an argon atmosphere for 1.5 h before cooling down to room temperature. The synthesized core-shell nanoparticles were collected by the addition of ethanol and washed for three times before dispersion in cyclohexane.

Synthesis of $NaGdF_4$:Yb/Tm@$NaYF_4$:20%Yb@$NaGdF_4$:10%Yb50%Nd and $NaGdF_4$:Yb/Tm@ $NaYF_4$:20%Yb@$NaGdF_4$:10%Yb50%Nd@$NaGdF_4$ (Gd-$CS_YS_2S_3$) core-multishell nanoparticles. The synthetic procedure for multi-shelled core-shell nanoparticles was identical to that for core-shell nanoparticles except for the use of a shell stock solution of $NaGdF_4$ with or without different composition of dopants. The preparation of other core-multishell nanoparticles including $NaGdF_4$:Yb/Tm@$NaGdF_4$:20%Yb@$NaGdF_4$:10%Yb50%Nd@$NaGdF_4$ (Gd-$CS_{Gd}S_2S_3$), $NaGdF_4$:Yb/Tm@ $NaYF_4$:20%Yb15%Tb@$NaGdF_4$:10%Yb50%Nd@$NaGdF_4$ (Gd-$CS_{Y\text{-}15\%Tb}S_2S_3$), $NaGdF_4$:Yb/Tm@$NaYF_4$: 20%Yb15%Eu@$NaGdF_4$:10%Yb50%Nd@$NaGdF_4$ (Gd-$CS_{Y\text{-}15\%Eu}S_2S_3$), $NaGdF_4$:Yb/Tm@$NaGdF_4$:20% Yb@$NaGdF_4$:10%Yb50%Nd@$NaGdF_4$:15%Tb (Gd-$CS_{Gd}S_2S_{15\%Tb}$), $NaGdF_4$:Yb/Tm@$NaGdF_4$:20%Yb@ $NaGdF_4$:20%Yb80%Nd@$NaGdF_4$:15%Tb (Gd-$CS_{Gd}S_{20\%Yb80\%Nd}S_{15\%Tb}$), $NaGdF_4$:Yb/Tm@$NaGdF_4$:20%Yb@ $NaYF_4$:10%Yb50%Nd@$NaGdF_4$ (Gd-$CS_1S_YS_3$), $NaGdF_4$:Yb/Tm@$NaGdF_4$:20%Yb@$NaGdF_4$:10%Yb 50%Nd@$NaYF_4$ (Gd-$CS_{Gd}S_2S_Y$), and $NaGdF_4$:Yb/Tm@$NaGdF_4$:20%Yb@$NaGdF_4$:10%Yb0%Nd@$NaGdF_4$ (Gd-$CS_{Gd}S_{0\%Nd}S_3$) $NaGdF_4$:Yb/Tm@$NaGdF_4$:20%Yb40%Y@$NaGdF_4$:10%Yb50%Nd@$NaGdF_4$(Gd-$CS_{40\%Y}S_2S_3$), $NaGdF_4$:49%Yb,8%Tm@$NaYF_4$:20%Yb@$NaGdF_4$:10%Yb,50%Nd@$NaGdF_4$ (Gd-$C_{8\%Tm}S_YS_2S_3$), $NaGdF_4$:49%Yb,8%Tm@$NaGdF_4$:20%Yb@$NaGdF_4$:10%Yb,50%Nd@$NaGdF_4$ (Gd-

$C_{8\%Tm}S_{Gd}S_2S_3$) were identical to that for core-shell nanoparticles except for the use of different composition of dopants.

Synthesis of $NaGdF_4$:49%Y,1%Tm@$NaYF_4$@$NaGdF_4$:10%Y,50%Nd@$NaGdF_4$ core-multishell nanoparticles. Gd/Tm co-doped $NaYF_4$ nanoparticles were prepared according to a modified literature procedure[4]. $Gd(CH_3CO_2)_3$ (0.134 g; 0.4mmol), $Y(CH_3CO_2)_3$ (0.106 g; 0.392 mmol) and $Tm(CH_3CO_2)_3$ (0.002 g; 0.008 mmol) in a water stock solution (4 mL) were added in a 50 mL three-neck round-bottom flask charged with OA (10 mL) and ODE (10 mL) at room temperature. The mixture was heated to 150 ℃ and maintained at this temperature for 1 h before cooling down to room temperature. A sodium oleate powder (2.5 mmol) was then added to the resulted solution. The obtained mixture was then heated to 100 ℃ and maintain at this temperature under vacuum for 1 h, followed by the addition of an ammonium fluoride solid (4 mmol). Subsequently, the resulting mixture was slowly heated to 160 ℃ and maintained at this temperature for 1.5 h, and then degassed for 10 min. The reaction mixture was then heated to 320 °C under a nitrogen atmosphere and kept at this temperature for 30 min. Then, the obtained nanoparticles were precipitated by the addition of ethanol and collected by centrifugation. After washing with ethanol and cyclohexane for three times, the obtained products were dispersed in cyclohexane. The synthetic protocol of shell coating onto the $NaGdF_4$:49%Y,1%Tm core nanoparticles was identical to that for $NaGdF_4$:Yb/Tm@$NaYF_4$:20%Yb@$NaGdF_4$:10%Yb50%Nd@$NaGdF_4$ core-multishell nanoparticles. The synthesis of $NaGdF_4$:20%Yb,30%Y@$NaYF_4$, $NaGdF_4$:20%Yb,1%Tm,29%Y@$NaYF_4$, $NaYF_4$:20%Yb,1%Tm@$NaYF_4$, were identical to that for $NaGdF_4$:49%Y,1%Tm@$NaYF_4$ @$NaGdF_4$:10%Y,50%Nd@$NaGdF_4$ nanoparticles except for the use of different composition of dopants.

Supplementary References

1. Wang, F., Deng, R. & Liu, X. Preparation of core-shell $NaGdF_4$ nanoparticles doped with luminescent lanthanide ions to be used as upconversion-based probes. *Nat. Protoc.* **9,** 1634–1644 (2014).
2. Su, Q. et al. The effect of surface coating on energy migration-mediated upconversion. *J. Am. Chem. Soc.* **134,** 20849−20857 (2012).
3. Wang, S. et al. Comparative investigation of the optical spectroscopic and thermal effect in Nd^{3+}-doped nanoparticles. *Nanoscale* **11,** 10220–10228 (2019).
4. Shi, R. et al. Tuning hexagonal $NaYbF_4$ nanocrystals down to sub-10 nm for enhanced photon upconversion. *Nanoscale* **9,** 13739–13746 (2017).
